# A Feasibility Study of Ammonia Recovery from Coking Wastewater by Coupled Operation of a Membrane Contactor and Membrane Distillation

**DOI:** 10.3390/ijerph15030441

**Published:** 2018-03-03

**Authors:** Po-Hsun Lin, Ren-Yang Horng, Shu-Fang Hsu, Shiao-Shing Chen, Chia-Hua Ho

**Affiliations:** 1 Department of Safety, Health and Environmental Engineering, Ming Chi University of Technology, No. 84 Gungjuan Rd., Taishan Dist., New Taipei City 24301, Taiwan; 2 Material and Chemical Research Laboratories, Industrial Technology Research Institute, 321 Sec. 2, Kuang Fu Rd., Hsinchu 300, Taiwan; vincenthorng@itri.org.tw (R.-Y.H.); katehsu@itri.org.tw (S.-F.H.); hua@itri.org.tw (C.-H.H.); 3 Institute of Environmental Engineering and Management, National Taipei University of Technology, No.1, Sec. 3, Chung Hsiao E. Rd., Taipei 106, Taiwan; f10919@ntut.edu.tw

**Keywords:** ammonia, coking wastewater, hollow fiber membrane contactor, membrane distillation, PTFE hollow fiber

## Abstract

More than 80% of ammonia (NH_3_) in the steel manufacturing process wastewater is contributed from the coking wastewater, which is usually treated by biological processes. However, the NH_3_ in the coking wastewater is typically too high for biological treatment due to its inhibitory concentration. Therefore, a two-stage process including a hollow fiber membrane contactor (HFMC) and a modified membrane distillation (MD) system was developed and applied to reduce and recover NH_3_ from coking wastewater. The objectives of this paper are to evaluate different membrane materials, receiving solutions, and operation parameters for the system, remove NH_3_ from the coking wastewater to less than 300 mg N/L, which is amenable to the biological process, and recover ammonia solution for reuse. As a result, the polytetrafluoroethylene (PTFE) HFMC using sulfuric acid as a receiving solution can achieve a maximum NH_3_-N transmembrane flux of 1.67 g N/m^2^·h at pH of 11.5 and reduce NH_3_ in the coking wastewater to less than 300 mg N/L. The NH_3_ in the converted ammonium sulfate ((NH_4_)_2_SO_4_) was then recovered by the modified MD using ice water as the receiving solution to produce ≥3% of ammonia solution for reuse.

## 1. Introduction

Ammonia is a major pollutant introduced into natural water systems by industrial and agricultural wastewater dischargers. Excessive ammonia in surface water is considered as an important source of eutrophication, the enrichment of water by nutrients causing an accelerated growth of algae, which often result in red water bloom in the lake or ocean. Ammonia is also toxic to most fish species and vertebrates causing convulsions, coma and death due to damage of central nervous system [1]. High concentrations of ammonia are commonly present in wastewater from several industries, including electronic, petrochemical, pharmaceutical, fertilizer, food, steel manufacturing industries and so on. Removal of ammonia from these industrial effluents is an important challenge because environmental laws and regulations governing safe discharge levels are becoming more stringent.

In aqueous solution, ammonia occurs in two forms, represented as ammonium ions (NH_4_^+^) and volatile ammonia (NH_3_). Both solution temperature and pH affect the amount of ammonia that can be removed from aqueous solution. Increasing temperature favors the presence of volatile ammonia in the aqueous solution because the solubility of ammonia decreases with increasing temperature, resulting in a higher vapor pressure. Raising pH values of the aqueous solution also reverses the ammonia dissociation and produces more volatile ammonia (Equation (1)), which results in a better ammonia removal efficiency. In addition, at high pH circumstances, the vapor pressure of solutions containing ammonia is higher than that of water. Increasing the ammonia concentration in water significantly increase the vapor pressure of the solution:

(1) $${\text{NH}}_{3}+{\text{H}}_{2}\text{O}\overset{K_{B}}{⇌}{{\text{NH}}_{4}}^{+}+{\text{OH}}^{-}$$

Coking wastewater is the effluents discharged from coke plants where coal is converted into coke that is suitable for use for blast furnaces in the integrated iron and steel plants [2]. Large quantities of water are used for the quenching of hot coke and for washing gas generated from coke ovens. Coking wastewater thus generated during coal coking, coal gas purification, and by-product recovery is highly polluted and difficult to treat. The composition of the wastewater is complicated depending on the different types of coal and the operating conditions in the coke ovens. The wastewater contains high concentration of ammonia and other compounds such as phenols, aromatic nitrogenated compounds and polycyclic aromatic hydrocarbons (PAHs), sulfide, cyanide (CN^−^), thiocyanide (SCN^−^) and so on [2,3,4].

Methods used to remove ammonia from wastewater include traditional biological treatments, air stripping, chemical precipitation, ion exchange, adsorption, advanced oxidation processes, anaerobic ammonium oxidation (ANAMMOX), reverse osmosis, electrodialysis, hollow fiber membrane contactor (HFMC) and membrane distillation (MD) [2,5,6,7,8,9,10,11,12,13,14,15,16]. Among these treatment methods, biological treatment requires energy for aeration and large space, and cannot treat the wastewater containing concentrated ammonia. Other physical and chemical treatment processes require large amount of chemical addition and further treatments for the ammonia contained end products (i.e., concentrated ammonia wastewater or struvite). HFMC and MD have received much attention for removal of ammonia in recent years due to the potentially low energy requirement and large surface area that facilitates fast separation of the ammonia from the wastewater [17,18,19,20,21]. It can be especially beneficial for wastewater streams with a high temperature. Both HFMC and MD also have the potential to recycle the ammonia as ammonia solution to solve problems resulting from ammonia contained end products.

The concept of membrane contactor technology is the use of hydrophobic membranes, typically in a hollow fiber configuration across which volatile species can transfer [17]. The membrane in a contactor acts as a passive barrier and as a means of bringing two immiscible fluid phases into contact with each other without dispersion [22]. Recently, HFMCs have been developed for gaseous component removal from aqueous solution, including ammonia [18,19,23], CO_2_ [24], and volatile organic compounds (VOCs) [25]. In ammonia removal process, an acid such as sulfuric acid is flowed counter-currently on the lumen side of the membrane to react with volatile ammonia to create ammonium sulfate ((NH_4_)_2_SO_4_). This process ensures that the difference in ammonia partial pressure on both sides of the membranes remains significant and thus provides the chemical potential that drives the separation process [23]. The method of using a microporous hydrophobic membrane to remove volatile species from solution into an acid wherein the gas can undergo a chemical reaction is generically termed Perstraction with Chemical Reaction (PCR) [26].

Being different from a membrane contactor, the MD process is a thermally driven process in which only vapor molecules are transported through a hydrophobic microporous membrane which acts as a barrier to physically separate the feed solution (warm side) from the permeate (cool side) which contains either a liquid or a gas phase. The hydrophobic microporous membrane prevents liquid solution from entering the pores by surface tension force, and a vapor-liquid interface is formed at the pore entrances where a vapor pressure gradient is generated by the concentration and temperature difference. As a result a driving force produced by the pressure gradient can drive vapor molecules of more volatile compounds to migrate from the feed to the permeate side of the membrane. At the permeate side, the migrated molecules are either condensed or removed in vapor/liquid phase out of the membrane module [20,27,28]. Generally, four common MD configurations include direct contact MD (DCMD), air gap MD (AGMD), sweep gas MD (SGMD), and vacuum MD (VMD). These MD configurations have been extensively reviewed and can be found elsewhere [18,20,27,29,30,31,32].

From the published literatures that applied MD to remove ammonia from water, Ding et al. compared the separation performance of VMD, SGMD, and DCMD used in the removal of ammonia from water in terms of mass transfer coefficient (K_a_) and selectivity (β) [21]. It was found that the order of K_a_ values are VMD > DCMD > SGMD; the order of β values are DCMD > SGMD > VMD. Factors such as membrane characteristics, feed temperature, permeate velocity, and the initial concentration and pH of the feed may affect the separation process. Tan et al. applied DCMD with polyvinylidene fluoride (PVDF) hollow fiber membranes for ammonia removal [33]. It has been indicated that the efficiency of ammonia removal is remarkably increased as the water pH is raised to 10, after which it gives no noticeable effect. In the study reported by EL-Bourawi et al., VMD equipped with polytetrafluoroethylene (PTFE) flat sheet membrane was employed for ammonia removal [27]. Within the operating parameters investigated such as feed temperature, flow velocity, and pH levels, and downstream pressure, the feed pH is the most dominant factor. Experimental results showed that high feed temperatures, low downstream pressures and high initial feed concentrations and pH levels enhance ammonia removal efficiency. Hasanoglu et al. used PTFE flat sheet and polypropylene (PP) hollow fiber membranes as the DCMD membrane modules to remove ammonia from water [18]. The circulation configuration and temperature of the feed solutions were found to have strong effect on the efficiency of the process.

**Figure 1:**
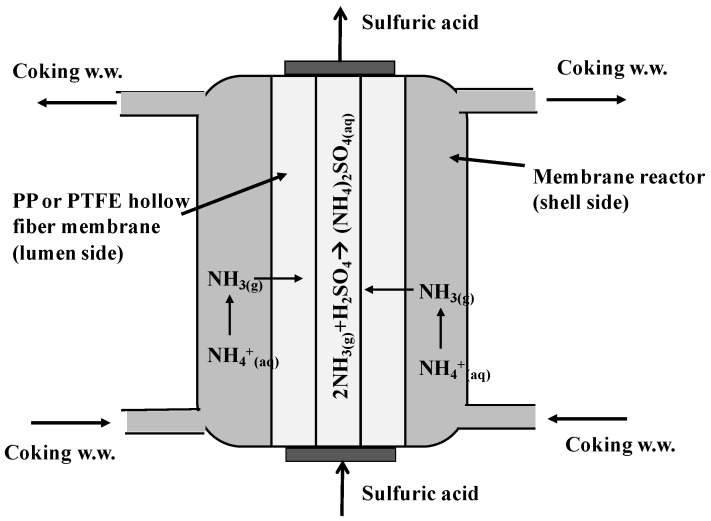
Principle of ammonia removal by hollow fiber membrane contactor (HFMC) module.

In this research, a two-stage process including a HFMC and a modified MD system was developed to remove ammonia from coking wastewater sampled from a steel manufacturing company in Taiwan. The first stage HFMC process was used as a pretreatment to remove ammonia to less than 300 mg/L for further biological treatments. Hydrophobic PP and PTFE hollow fiber membranes were employed in the HFMC module to evaluate the membrane feasibility. The detailed principle of the ammonia removal in the HFMC is illustrated in Figure 1. The volatile ammonia (NH_3_) diffuses across the membrane and reacts immediately with sulfuric acid (used as a receiving solution) on the interface to form nonvolatile compound, ammonium sulfate ((NH_4_)_2_SO_4_). Ammonium sulfate is commonly used as a soil fertilizer for crop production. However, the ammonium sulfate produced from industrial waste and wastewater is considered as hazardous waste and is not allowed to reuse by fertilizer companies in Taiwan. This results in the difficulty to handle ammonium sulfate. Therefore, we developed another modified MD system as a second stage process to recover ammonia from concentrated ammonium sulfate solution produced from the HFMC to form ammonia solution that can be reused in the coke plant. The issue of ammonium sulfate waste can also be solved. The modified MD system, differing from conventional MD systems, has potential to prevent membrane fouling and wetting, which are two potential problems that may lead to MD operation failures. In the problem of membrane fouling, the foulants attach onto the membrane surface, block the pores, and consequently reduce vapor flux [34]. For membrane wetting, the presence of high organics or surfactant in the feed solution can lower the surface tension of the feed and/or reduce the hydrophobicity of the membrane and lead to wetting [35]. To the best of our knowledge, this study is the first research that applied real coking wastewater as the wastewater source to evaluate the feasibility of ammonia recovery by coupled operation of the HFMC and modified MD process.

## 2. Materials and Methods 

### 2.1. HFMC Module

**Figure 2:**
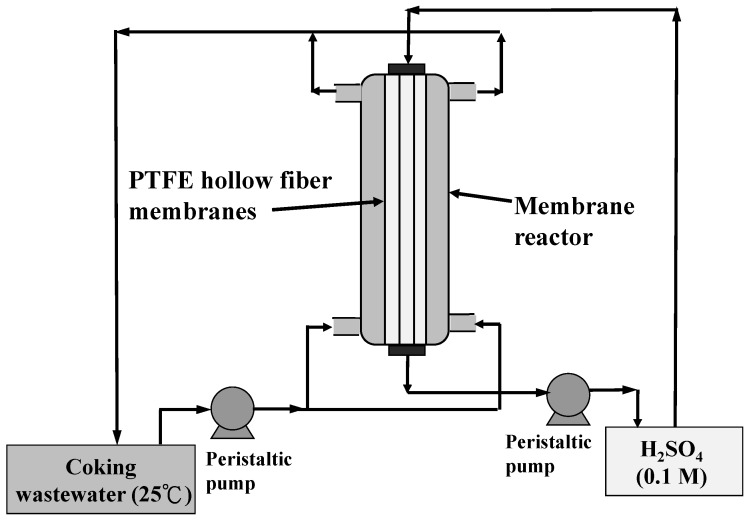
Schematic of the HFMC apparatus used at the 1st stage ammonia removal.

The HFMC module used for ammonia removal is shown schematically in Figure 2. Commercial hydrophobic PP and PTFE hollow fiber membranes supplied by Century Environtech Co., (New Taipei City, Taiwan), were employed in this study. Structural parameters of the PP and PTFE hollow fiber membranes are listed in Table 1. Diluted sulfuric acid (0.1 M) were used as receiving solution. The feed coking wastewater was circulated through the shell side of the membrane reactor by a peristaltic pump, while the receiving solution was circulated into the lumen side in a countercurrent flow by another peristaltic pump. Both feed and receiving solutions were recycled to their reservoirs with volumes of 2000 and 500 mL, respectively. The temperature of the feed solution was maintained at room temperature (25 °C). The desired pHs of coking wastewater were adjusted by addition of sodium hydroxide (NaOH). During the experiment runs, the concentrations of ammonia (NH_3(aq)_) in the coking wastewater and receiving solution were monitored using an ion-selective electrode (IntelliCAL™ ISENH4181, HACH, Loveland, CO, USA)

### 2.2. Modified MD Module

**Figure 3:**
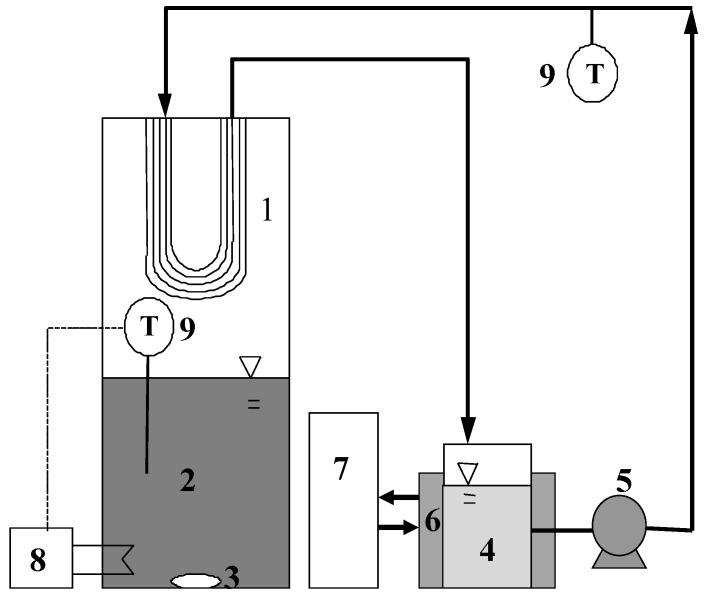
Schema of the modified MD apparatus employed (1: PTFE hollow fiber membrane; 2: feed solution ((NH_4_)_2_SO_4_); 3: magnetic stirrer; 4: ammonium hydroxide solution; 5: peristaltic pump; 6: ice water bath (10 °C); 7: chiller; 8: thermostatic bath (40 °C); 9: thermometers).

The modified MD was supplied by the Industrial Technology Research Institute (ITRI) in Taiwan, and the experimental setup is illustrated in Figure 3. Unlike conventional MD modules, the PTFE membrane in the modified MD module was suspended on the top of the membrane module and did not directly contact with the feed solution (i.e., the concentrated (NH_4_)_2_SO_4_ generated by the HFMC module). The temperature of the feed solution was maintained at 40 °C, while the pH was raised to ≥11 to convert NH_4_^+^ to volatile NH_3_ that can provided driving force for permeation. Ice deionized water (10 °C) generated by a chiller was used as the receiving solution to circulate into the lumen side by a peristaltic pump, and the permeate NH_3_ vapor was dissolved in the ice water to form ammonia solution, which can be reused in the coke plant.

### 2.3. Characteristics of Coking Wastewater

Coking wastewater was collected six times from a steel manufacturing company. The main characteristics of the coking wastewater used as HFMC feed solutions during the study period are summarized in Table 2. Chemical oxygen demand (COD) was measured by the AQUAfast COD colorimeter (Orion AQ2040, Thermo Scientific, Waltham, MA, USA).

## 3. Results and Discussion

### 3.1. Evaluation of HFMC Membrane Materials and Receiving Solutions

#### 3.1.1. PP Hollow Fiber Membranes

**Figure 4:**
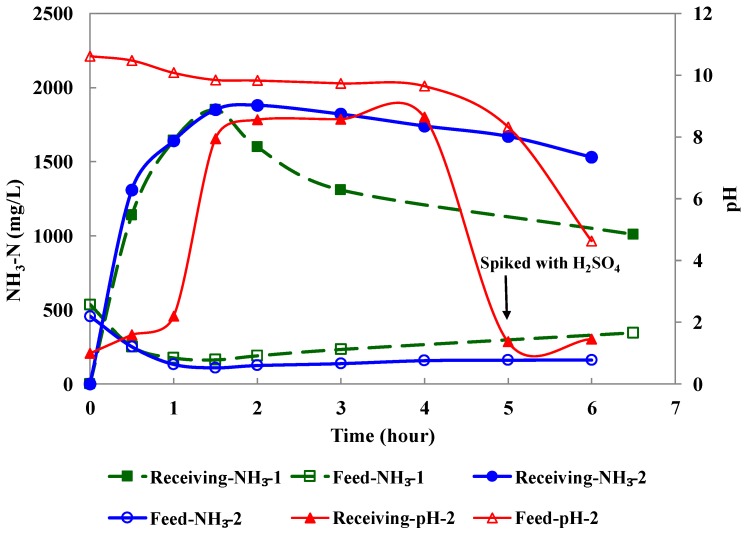
The NH_3_-N concentration in the feed and receiving solution as a function of time using PP hollow fiber membranes.

PP hollow fiber membranes were evaluated to remove ammonia from coking wastewater as the feed solution in the HFMC module. Sulfuric acid (0.1 M) was used as the receiving solution. The pH of coking wastewater was raised to 10.7. The circulation flow rate of the feed and receiving solutions were 410 and 110 mL/min, respectively. Figure 4 shows representative results from the duplicate experiments in which a new PP membrane module was tested. The initial NH_3_-N concentration in the coking wastewater was approximately 500 mg/L. At 1.5 h the NH_3_-N in the feed was reduced to a minimum level of ~150 mg/L (equal to 70% removal) and then slightly increased, while the NH_3_-N concentrations in the receiving solution reached a maximum level of 1850 mg/L and decreased afterward. In the second experiment, the pH of the receiving solution increased from 1 to 8 at the 1.5th h while the pH of the feed solution decreased from 10.7 to 9.8 simultaneously. Sulfuric acid was spiked into the receiving solution at the 5th h which resulted in a significant decrease of pH. Meanwhile, the pH of the feed solution declined after the 5th h as well. The agreement of the declined NH_3_-N concentrations, increased pH in the receiving solution, and decreased pH in the feed solution indicated that the PP membranes were wetted after 1.5 h operation in the second test. This result indicated that PP membranes were not applicable for HFMC module to remove ammonia from coking wastewater.

#### 3.1.2. PTFE Hollow Fiber Membranes

**Figure 5:**
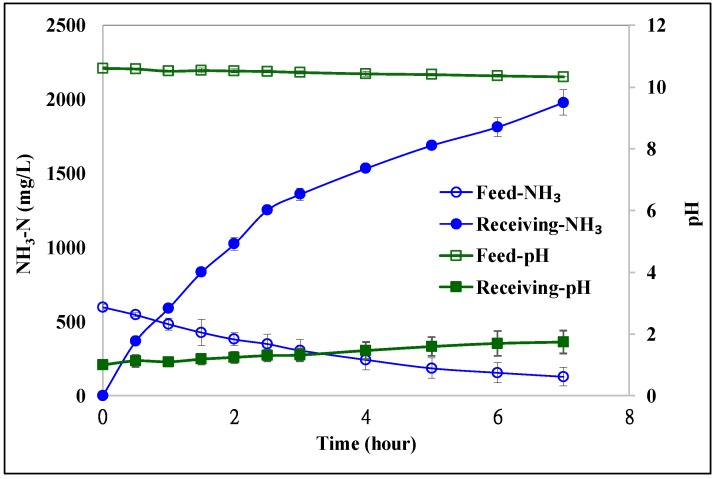
The NH_3_-N concentrations and pH in both feed and receiving solutions as a function of time using PTFE hollow fiber membranes (receiving solution: 0.1 M H_2_SO_4_).

PTFE hollow fiber membranes were applied in the HFMC module to remove ammonia from coking wastewater. 0.1 M H_2_SO_4_ solution was used as the receiving solution, and the pH of coking wastewater was adjusted to 10.7. The circulation flow rate of the feed and receiving solutions were 590 and 250 mL/min, respectively. Figure 5 shows the NH_3_-N concentrations and pH in both feed and receiving solutions as a function of time. Two replicate experiments were conducted. The NH_3_-N in the feed was reduced from 610 to 255 mg/L (equal to 58% removal) at the 3rd h, and reached to 85 mg/L (equal to 86% removal) at the 7th h, while the NH_3_-N in the receiving solutions was concentrated to 1900 mg/L. The NH_3_-N transmembrane flux for the first 3 h was calculated to be 1.2 g/m^2^·h. Unlike PP membranes, the pH of the feed and receiving solution at the 7th h only slightly changed to 10.3 and 2, respectively, which indicated that the PTFE membranes were feasible for the HFMC module to remove ammonia from coking wastewater.

PP and PTFE are the most common materials used for MD membranes. PTFE membranes have lower reported surface energy (ranging from 9–20 × 10^−3^ N/m) than that of PP membranes (30.0 × 10^−3^ N/m), which results in higher hydrophobicity of PTFE then PP membranes [35]. In addition, coking wastewater could contain high concentrations of complicated organic matters such as hydrophobic, hydrophilic, and amphiphilic organics that can reduce the hydrophobicity of the membrane by adsorption and lead to membrane wetting. The lower hydrophobicity of PP membranes and high organic content of coking wastewater can explain that PP membranes are infeasible in HFMC module contacting with coking wastewater.

#### 3.1.3. Evaluation of Distilled Water as the Receiving Solution

**Figure 6:**
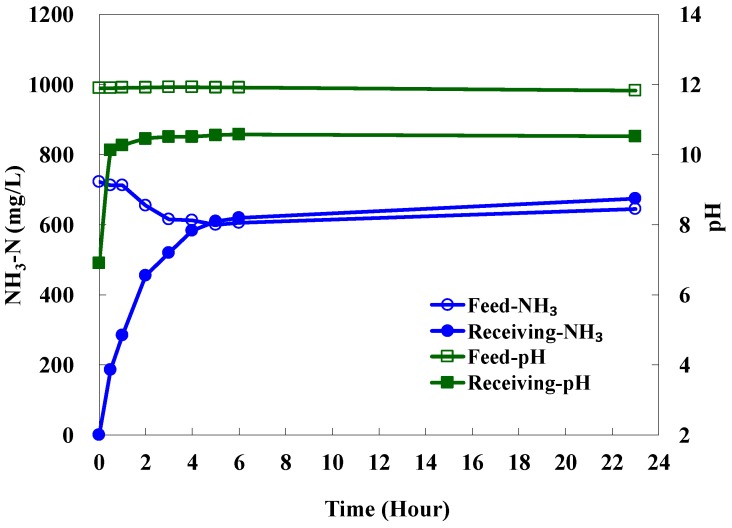
The NH_3_-N concentrations and pH in both feed and receiving solutions as a function of time using PTFE hollow fiber membranes (receiving solution: distilled water, 10 °C).

Cool distilled water (10 °C) was evaluated its applicability as the receiving solution in the HFMC module using PTFE hollow fiber membranes to remove ammonia from coking wastewater. The pH of coking wastewater was raised to 11.9. The circulation flow rate of the feed and receiving solutions were 900 and 830 mL/min, respectively. The NH_3_-N concentrations and pH in both feed and receiving solutions as a function of time are presented in Figure 6. The NH_3_-N concentration in the feed was reduced from 720 to 600 mg/L at the 6th h. Meanwhile the NH_3_-N in the distilled water reached 620 mg/L. The NH_3_-N transmembrane flux for the first 2 h was calculated to be 0.81 g/m^2^·h, lower than the flux using H_2_SO_4_ solution as the receiving solution. The experiment was continuously conducted for 23 h, during which the NH_3_-N in the feed and receiving solution maintained at a stable level. This indicated that the equilibrium state of NH_3_-N had been attained since the 5th h. The pH of the feed solution during the entire experiment was stabilized at 11.8, while the pH of the receiving solution increased from 7 to 10.5 at the 2nd h and retained to the end of the experiment. The result indicated that H_2_SO_4_ solution is a preferable receiving solution in the HFMC module because the reaction rate and solubility of ammonia with H_2_SO_4_ solution is greater than that with cool water. The pH result also demonstrated that the chemical property of PTFE membranes were applicable for HFMC modules to remove ammonia from coking wastewater.

### 3.2. Effects of Feed pH Level for the PTFE HFMC Module

**Figure 7:**
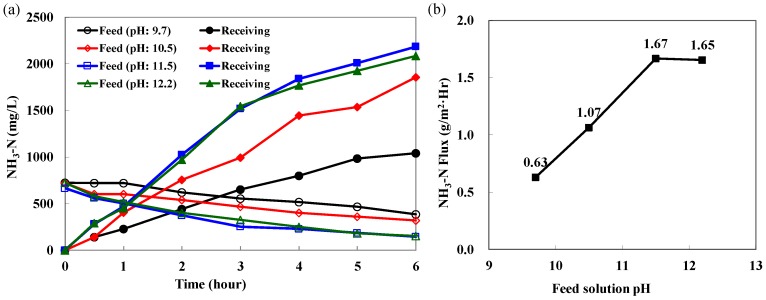
(**a**) the NH_3_-N concentrations in both feed and receiving solutions as a function of time; (**b**) the NH_3_-N transmembrane flux as a function of pH.

As described above, the PTFE HFMC module using H_2_SO_4_ solution as the receiving solution shows excellent reliability to remove ammonia from the coking wastewater. Different pH levels of the feed solution for the PTFE HFMC module were evaluated to optimize the HFMC operation. Increasing pH values of the solution favors the presence of volatile ammonia and is very likely to increase the NH_3_-N flux in the HFMC module. The initial NH_3_-N concentration in the coking wastewater was 740 mg/L. The temperature of the coking wastewater was controlled at room temperature (25 °C). Four pH values, 9.7, 10.5, 11.5, and 12.5, were tested, and the experimental parameters are listed in Table 3. The pH of the feed solution nearly maintained the initial pH, and the pH of the receiving solution slightly increased by one pH unit, which is similar to the pH results presented in Figure 5. The NH_3_-N concentrations in both feed and receiving solutions as a function of time are presented in Figure 7a. For the initial pH of 11.5 and 12.5, the NH_3_-N concentrations were reduced to 225 and 250 mg/L at the 4th h, achieving the objective of less than 300 mg/L, and then decreased to 150 mg/L at the 6th h, equal to 80% removal, while for the initial pH of 9.7 and 10.5, the NH_3_-N concentration in the feed remained 520 and 400 mg/L, respectively. The averaged NH_3_-N transmembrane flux from the membrane to the receiving solution for the first 3 h as a function of pH is shown in Figure 7b. As it is expected, raising pH of the coking wastewater increased the ammonia removal efficiency and NH_3_-N transmembrane flux. The NH_3_-N was directly proportional to the feed pH up to a pH of 11.5 and then reached a plateau because the distribution of volatile ammonia in the solution at pH ≥ 11.5 is very similar. In this case, adjusting the feed pH ≥ 11.5 resulted in the highest NH_3_-N transmembrane flux. The experiment also indicated that ammonia separation process by MD was remarkably affected by pH adjustment.

### 3.3. Ammonia Solution Recovered by the Modified MD Module

#### 3.3.1. Accumulation of Concentrated (NH_4_)_2_SO_4_ Solution

The (NH_4_)_2_SO_4_ solution produced from the coking wastewater is considered as hazardous waste and requires further treatment by waste disposing services. The PTFE HFMC module was used to concentrate the (NH_4_)_2_SO_4_ solution for further treatment to solve the (NH_4_)_2_SO_4_ waste issue. In this experiment, the pH of coking wastewater was raised to 11.5, and the circulation flow rate of feed and receiving solution was 830 mL/min. 

**Figure 8:**
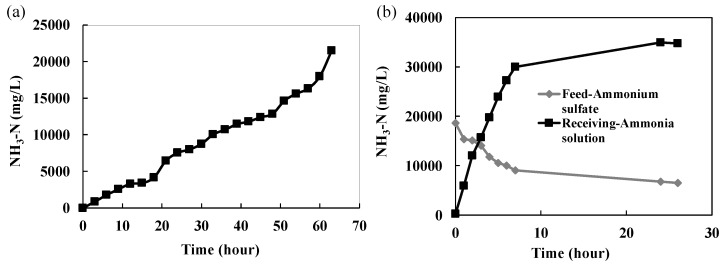
(**a**) Ammonia accumulation in (NH_4_)_2_SO_4_ solution as a function of time; (**b**) The NH_3_-N concentrations in both feed and receiving solutions as a function of time using the modified MD (receiving solution: 10 °C ice deionized water).

The pH of the receiving solution was maintained at 1–2. As the NH_3_-N concentration in the coking wastewater was decreased to less than 300 mg/L, another batch of coking wastewater was replaced until the NH_3_-N in the (NH_4_)_2_SO_4_ solution achieved ≥20,000 mg/L. Figure 8a shows the NH_3_-N concentration in the (NH_4_)_2_SO_4_ solution as a function of time, in which 22 batches of coking wastewater were treated. After 66 h, the NH_3_-N concentration in the (NH_4_)_2_SO_4_ solution reached 25,000 mg/L and can even achieve a higher concentration.

#### 3.3.2. Ammonia Solution Recovery

The concentrated (NH_4_)_2_SO_4_ solution was placed in the modified MD for ammonia separation and recovery. The pH and temperature of the (NH_4_)_2_SO_4_ solution was adjusted to 11.8 and 40 °C, respectively. Ice deionized water (10 °C) was used as the receiving solution to adsorb the permeated NH_3_ vapor to form ammonia solution. The NH_3_-N concentrations in both feed and receiving solutions as a function of time are presented in Figure 8b. The NH_3_-N concentration in the (NH_4_)_2_SO_4_ solution was ~19,000 mg/L in this test and was reduced to 9000 mg/L at the 7th h, while the NH_3_-N in the ammonia solution was increased linearly to 30,000 mg/L at the initial 7 h and was considered in equilibrium with its vapor phase afterwards. The NH_3_-N transmembrane flux for the first 7 h was 20 g/m^2^·h. The 3% ammonia solution can be reused in the coke plant, while the residual (NH_4_)_2_SO_4_ solution could be mixed with the concentrated (NH_4_)_2_SO_4_ solution produced from the 1st stage HFMC or used as the receiving solution in the HFMC, in which the pH should be lowered to ≤1.

For the four common MD configurations, including DCMD, AGMD, VMD and SGMD, the membranes are in direct contact with feed liquid, and the configurations differed base on the nature of the cold side processing of the permeate. In these traditional MD configurations, membrane fouling, scaling, and wetting are major limitations for applying MD in industries. Unlike conventional MD configurations in which membranes are directly contacted with feed solutions, the membranes in the modified MD module developed in this study did not directly contact with the feed solution. It has the potential to prevent membrane fouling, scaling, and wetting and extend the membrane life span. Moreover, it is beneficial to apply to feed water with complex properties and/or with concentrated acid, base, or organics that could readily damage and/or wet the membranes.

#### 3.3.3. Economic Analysis

The major cost of this ammonia removal and recovery system is chemical cost, including alkali (NaOH) and acid (H_2_SO_4_) addition since heat and ice water can be obtained by energy integration in the steel manufacturing company with lower cost. According to the experimental results, we speculated that for the first stage HFMC system, 5 L of 45% NaOH and 0.8 L of 50% H_2_SO_4_ are required to treat one m^3^ of the coking wastewater. The cost is around 35 NT$/m^3^ (i.e., ~1.17 US$/m^3^. For the second stage modified MD system, 2.2 L of 45% NaOH is required to recover 3% ammonia solution, which is equal to 14 NT$/m^3^ (i.e., ~0.47 US$/m^3^). Therefore, the total chemical cost is approximate 49 NT$/m^3^ (i.e., ~1.64 US$/m^3^). Furthermore, at least 5 L of 3% ammonia solution can be recovered by treating one m^3^ of the coking wastewater.

## 4. Conclusions

A two-stage processes including a HFMC and a modified MD system developed in this research was capable of reducing and recycling ammonia from coking wastewater producing via steel manufacturing processes. The experimental results presented confirm that the PTFE hollow fiber membranes were more feasible than PP membranes for the HFMC system to remove ammonia from coking wastewater due to the higher hydrophobicity of PTFE membranes. Comparing with cool water, H_2_SO_4_ solution is a preferable receiving solution in the HFMC module because the reaction rate and solubility of ammonia with H_2_SO_4_ solution is greater than that with cool water. Since pH significantly affects the distribution of ammonia and ammonium ion in water, raising pH of the coking wastewater increased the ammonia removal efficiency and NH_3_-N transmembrane flux. It is concluded that PTFE HFMC module using sulfuric acid as a receiving solution can achieve a maximum NH_3_-N flux of 1.67 g N/m^2^·h at pH of 11.5 and reduce NH_3_ in the coking wastewater to less than 300 mg N/L for further biological treatment processes. Meanwhile, the HFMC module can concentrate the (NH_4_)_2_SO_4_ solution to ≥20,000 mg/L by replacing several batches of raw coking wastewater. The NH_3_ in the ((NH_4_)_2_SO_4_) was then recovered by the modified MD using ice water as the receiving solution to produce ≥3% of ammonia solution for reuse in the coke plant. At least 5 L of 3% ammonia solution can be recovered by treating one m^3^ of the coking wastewater. The residual (NH_4_)_2_SO_4_ solution could be mixed with the concentrated (NH_4_)_2_SO_4_ solution produced from the 1st stage HFMC or used as the receiving solution in the HFMC, in which the pH should be lowered to ≤1.

## Figures and Tables

**Table 1:** Structural parameters of the polypropylene (PP) and polytetrafluoroethylene (PTFE) hollow fiber membranes.

Parameter, Unit	PP	PTFE
Pore diameter (µm)	0.1–0.3	0.2–0.3
Membrane thickness (µm)	45	600
Outer diameter (mm)	0.4	2.2
Surface area (m^2^)	0.64	0.15
Porosity	Not available	0.53

**Table 2:** Characteristics of coking wastewater.

COD (mg/L)	pH	NH_3_-N (mg/L)	Conductivity (μs/cm)	Phenol (mg/L)	CN^−^ (mg/L)	SCN^−^ (mg/L)
5211 ± 640	5.5–8.2	648 ± 158	6495 ± 895	945 ± 168	2.5 ± 0.2	512 ± 55

**Table 3:** Experimental parameters of the PTFE HFMC modules that varied the feed pH.

	Feed Solution	Receiving Solution
Component	Coking wastewater	0.1 M H_2_SO_4_
Volume (mL)	2000	500
Initial pHs	9.7, 10.5, 11.5, 12.5	<1
Circulation flow rate (mL/min)	830	830
